# Effect of glycemic state on postprandial hyperlipidemia and hyperinsulinemia in patients with coronary artery disease

**DOI:** 10.1007/s00380-015-0757-y

**Published:** 2015-10-06

**Authors:** Akihiro Nakamura, Yuto Monma, Shoko Kajitani, Kazuki Noda, Sota Nakajima, Hideaki Endo, Tohru Takahashi, Eiji Nozaki

**Affiliations:** Department of Cardiology, Iwate Prefectural Central Hospital, 1-4-1 Ueda, Morioka, 020-0066 Japan

**Keywords:** Diabetes, Postprandial hyperlipidemia, Hyperinsulinemia, Coronary artery disease, Insulin resistance

## Abstract

Both postprandial hyperlipidemia and hyperinsulinemia have been thought to play an important role in the development of atherosclerosis, and to be a potent risk factor for cardiovascular event. To examine effects of glycemic state on postprandial hyperlipidemia and hyperinsulinemia in patients with coronary artery disease (CAD), a total of 112 consecutive male pati ents with angiographically confirmed CAD were loaded with a high-fat and high-glucose test meal. CAD patients were divided into three groups as “non-diabetic”, “prediabetic”, and “diabetic” CAD groups. The serum triglyceride (TG) and remnant-like particle cholesterol (RLP-C) levels at the 6th hour in diabetic CAD group showed significantly higher than non-diabetic CAD group, and the incremental area under the curves (*i*AUCs) of these levels in diabetic CAD group were significantly greater than non-diabetic CAD group (TG, *P* = 0.0194; RLP-C, *P* = 0.0219). There were no significant differences in the *i*AUCs of TG or RLP-C between prediabetic and non-diabetic CAD group. The AUCs of plasma insulin levels or insulin resistance index (IRI): (AUCs of insulin) × (AUCs of glucose) as the insulin resistance marker were greater in diabetic CAD group than non-diabetic CAD group (insulin, *P* = 0.0373; IRI, *P* = 0.0228). The AUCs of serum TG or RLP-C levels showed a correlation with the AUCs of plasma insulin (AUC-TG, *r* = 0.5437, *P* < 0.0001; AUC-RLP-C, *r* = 0.6847, *P* < 0.0001), and they correlated well with the insulin resistance index (AUC-TG, *r* = 0.7724, *P* < 0.0001; AUC-RLP-C, *r* = 0.7645, *P* < 0.0001). We found that the insulin resistance showed a close relationship with postprandial hyperlipidemia in CAD patients. Diabetic, but not prediabetic state, may be a risk for postprandial impaired lipid metabolism in CAD patients.

## Introduction

The importance of hyperlipidemia, especially elevated serum levels of low-density lipoprotein cholesterol (LDL-C), has been established as a risk factor for cardiovascular disease [1]. Although the direct effect of serum triglyceride (TG) on atherosclerotic lesion formation has been still unclear, recent meta-analysis and epidemiologic studies have revealed that elevated serum levels of TG are associated with the development of coronary artery disease (CAD) independent of other coronary risk factors [2, 3]. Postprandial hyperlipidemia characterized by pronounced and prolonged serum levels of TG, has been shown to play an important role in progression or vulnerability of coronary arterial plaque [4]. Not only postprandial hyperlipidemia but also hyperinsulinemia has been reported as the metabolic condition that could predispose individuals to the development of atherosclerosis and CAD [5, 6].

Although type 2 diabetes mellitus (T2DM) is a well-known potent risk factor for the development of CAD, little is known the relationship between postprandial lipid dysmetabolism and glycemic state in patients with CAD. In the present study, we examined whether a significant correlation existed between the severity of glycemic state and the degree of postprandial lipidemic responses for studying the impact of diabetic condition on postprandial hyperlipidemia or hyperinsulinemia in patients with CAD.

## Methods

### Study patients

A total of 112 consecutive male patients with stable angina pectoris (mean age 66.8 ± 7.9 years) who were angiographically confirmed CAD and fulfilled the following exclusion criteria, were loaded with a high-fat and high-glucose test meal at Iwate Prefectural Central Hospital from January 2013 to June 2014: (1) body mass index (BMI) > 25.0; (2) T2DM with insulin therapy; (3) gastrointestinal disease limiting drug absorption or partial ileal bypass; (4) major surgery within 6 months prior to enrollment or concomitant inflammatory disease or malignant tumors; (5) congestive heart failure or active liver disease or hepatic dysfunction defined as alanine aminotransferase or aspartate aminotransferase > normal range; (6) concurrent therapy with long-term immunosuppressants; (7) familial hypercholesterolemia; (8) taking lipid lowering medications without statins (e.g., ezetimibe, cholestyramine, niacin, or fibrates) and/or eicosapentaenoic acid or docosahexaenoic acid therapy.

### Ethics

This study was conducted according to the principles expressed in the Declaration of Helsinki. Written informed consent was obtained from the subjects and the study design was approved by the ethics committee of the Iwate Prefectural Central Hospital.

### Definitions

Patients with stable angina pectoris were defined as cardiac ischemic patients who had a history of myocardial infarction, coronary artery bypass, percutaneous coronary intervention with or without stenting, or previous angiographically proved stenotic lesion ≥75 % in a major epicardial coronary artery. They were also diagnosed as stable condition when chest pain was brought on by exertion, resolved under nitrate-therapy and not changed in its characteristics (frequency, severity, duration, time of appearance and precipitating factors) for the previous 60 days [7].

Patients with CAD were classified into three groups (non-diabetic, prediabetic and diabetic groups) according to glycated hemoglobin A1c (HbA1c) levels and fasting plasma glucose (FPG) levels in accordance with 2010 American Diabetes Association (ADA) Guidelines [8, 9]. Non-diabetic patients were defined as patients without dysglycemia which was indicated by the presence of either T2DM or prediabetes. Diagnosis of T2DM was made according to ADA criteria of a FPG level of ≥126 mg/dL, HbA1c ≥ 6.5 %, or current use of hypoglycemic agents. Prediabetes was indicated by a FPG level of 100–125 mg/dL or a HbA1c level of 5.7–6.4 % without T2 DM.

### Study design

For fat loading, all patients were given an oral high-fat and high-glucose meal for breakfast after overnight fasting for at least 12 h (named as cake sále test). This cake sále consisted of high-fat and high-glucose [1003 kcal; protein, 28.6 g (11.4 %); lipids, 62.4 g (56.0 %); carbohydrate, 80.7 g (32.2 %); cholesterol, 320.5 mg (0.4 %)] and its ingredients were similar to those of an American fast-food meal (Big Mac Cheeseburger^®^ with French fries, Coca-Cola^®^) which was one of the most popular food in the world [10]. Patients were requested to eat this high-fat and high-glucose meal (cake sále) in 30 min. In all patients, this cake sále test was performed in stable condition. Blood samples were obtained by venipuncture during the fasting state just before the cake sále test and 0, 2, 4, and 6 h after the test. Sera were separated immediately after blood collection by low-speed centrifugation (at 3000 rpm for 15 min at 4 ºC) and stored at −80 ºC until measurements. Serum levels of total cholesterol (TC) and TG were determined by enzymatic methods, serum LDL-C and high-density lipoprotein cholesterol (HDL-C) levels by a direct method, serum malondialdehyde-modified LDL (MDA-LDL) levels by a sandwich ELISA method, serum apolipoprotein A-I (Apo A-I) and apolipoprotein B (Apo B) levels by an immunoturbidity method, and serum remnant-like particle cholesterol (RLP-C) levels by the immunoaffinity isolation method, respectively, at a contract laboratory (SRL Co., Ltd., Tokyo, Japan). Serum LDL-C levels were determined by direct measurement and not by calculation by the Friedewald formula, since the postprandial TG levels were predicted to 400 mg/dL.

Plasma glucose and insulin levels were also measured for the assessment of glucose metabolism before and after a meal load. Plasma insulin levels were determined by a chemiluminescent enzyme immunoassay method, HbA1c levels by a high-performance liquid chromatography method, respectively, at the laboratory of our hospital.

Each fasting values were obtained before the cake sále test. All samples were treated in accordance with the Helsinki Declaration.

### Insulin resistance markers

In the present study, the following insulin resistance makers were measured: (1) fasting insulin levels, (2) insulin levels at 2 h after the cake sále test, (3) the area under the curve (AUC) of insulin during the test, (4) the insulin resistance index: [(AUCs of insulin) × (AUCs of glucose)], (5) the homeostasis model assessment of insulin resistance (HOMA-IR). HOMA-IR was calculated as [fasting plasma glucose (mg/dL) × fasting plasma insulin (μIU/mL)]/405 [11, 12]. The AUC was calculated by the trapezoidal method, and incremental AUC (*i*AUC) was calculated as total AUC minus the area under the basal value.

### Statistical analysis

All values are expressed as mean ± standard deviation for continuous variables and as numbers and percentages for categorical variables. Differences between two groups were assessed using Student’s unpaired *t* test or Mann–Whitney’s *U* test for continuous variables and Chi-square test or Fisher’s exact test for categorical variables, as appropriate. One-way analysis of variance followed by Tukey–Kramer honestly significant difference test was used to examine differences among multiple groups. Correlation between the two parameters was determined by simple linear regression analysis. A two-sided *P* value of less than 0.05 was considered statistically significant. All statistical analyses were performed with SPSS version 14.0 (SPSS Inc., Chicago, IL, USA).

## Results

### Patient characteristics

Table 1 shows the baseline characteristics of the patients (*n* = 112) divided into three groups: 32 patients (29 %) in non-diabetic CAD group; 47 patients (42 %) in prediabetic CAD group; 33 patients (29 %) in diabetic CAD group. The levels of HbA1c and FPG were 5.4 ± 0.2 %, 90.5 ± 14.3 mg/dL in non-diabetic CAD group; 6.0 ± 0.2 %, 103.8 ± 14.6 mg/dL in prediabetic CAD group; 7.1 ± 0.8 %, 138.7 ± 9.6 mg/dL in diabetic CAD group, respectively. All patients had received statins, and no significant differences in lipid markers were observed such as TG, LDL-C, HDL-C, and RLP-C among three groups. Patients with BMI level >25.0 were excluded in the present study, and there was no significant difference in body size. There were also no significant differences in age, blood pressure, and the incidence of hypertension or smoking among three groups.

**Table 1:** Baseline characteristics in patients

Variable	Non-diabetic CAD group (*n* = 32)	Prediabetic CAD group (*n* = 47)	Diabetic CAD group (*n* = 33)	*P* value^#^
Age (years)	68.5 ± 6.5	66.1 ± 8.6	66.8 ± 8.0	0.8223
Weight (kg)	63.1 ± 4.9	61.0 ± 6.0	62.6 ± 8.1	0.7472
Height (cm)	163.3 ± 3.0	163.2 ± 4.9	166.5 ± 8.3	0.3463
BMI (kg/m^2^)	23.1 ± 1.4	22.9 ± 1.6	22.5 ± 2.1	0.7323
Hypertension (*n*) (%)	20 (63)	30 (64)	22 (67)	0.5864
*Blood pressure (mmHg)*				
Systolic	136.2 ± 8.1	127.6 ± 19.7	123.9 ± 17.1	0.2852
Diastolic	69.7 ± 12.8	77.4 ± 10.8	71.8 ± 15.2	0.4769
Current or ex-smokers (*n*) (%)	21 (66)	33 (70)	23 (70)	0.6271
*Glucose markers*				
HbA1c (%)	5.4 ± 0.2	6.0 ± 0.2	7.1 ± 0.8	<0.0001
Fasting plasma glucose (mg/dL)	90.5 ± 14.3	103.8 ± 14.6	138.7 ± 9.6	0.0002
Fasting plasma insulin (μIU/mL)	6.1 ± 3.1	6.4 ± 3.8	8.4 ± 3.7	0.1102
Fasting plasma C-peptide (ng/mL)	1.4 ± 0.5	1.8 ± 0.9	2.6 ± 0.5	0.0808
Use of stain (*n*) (%)	32 (100)	47 (100)	33 (100)	1.0000
*Lipid markers*				
Total cholesterol (mg/dL)	159.7 ± 31.4	171.6 ± 29.4	175.5 ± 22.1	0.3653
Triglyceride (mg/dL)	122.7 ± 40.5	141.2 ± 74.7	140.3 ± 76.8	0.9845
LDL cholesterol (mg/dL)	95.8 ± 27.9	100.5 ± 30.4	100.5 ± 20.1	0.9054
HDL cholesterol (mg/dL)	46.5 ± 11.5	51.9 ± 12.7	55.6 ± 16.8	0.3710
RLP cholesterol (mg/dL)	4.3 ± 1.3	4.9 ± 2.5	4.9 ± 2.6	0.6630
MDA LDL (U/L)	98.7 ± 36.0	107.3 ± 28.6	114.3 ± 32.6	0.4975
Apolipoprotein A-I (mg/dL)	121.7 ± 19.0	137.5 ± 26.6	139.7 ± 30.9	0.3799
Apolipoprotein B (mg/dL)	82.5 ± 18.8	85.0 ± 22.4	86.9 ± 15.7	0.8439

Values for continuous variables are shown as mean ± SD; categoric variables are represented by number (percentage, %)

*BMI* body mass index, *HbA1c* hemoglobin A1c, *LDL* low-density lipoprotein, *HDL* high-density lipoprotein, *RLP* remnant lipoprotein, *MDA* malondialdehyde-modified

^#^Difference among three groups analyzed by one-way analysis of variance (ANOVA) for continuous variables, chi-square test for categoric variables

### Postprandial changes of lipid and glucose markers

The changes of lipid and glucose markers in the cake sále test are summarized in Table 2. In all the three groups, the serum TG and RLP-C levels showed the significant changes during the test. Other lipid markers did not show any meaningful changes from the baseline. There were also significant changes in plasma glucose and insulin levels during the test in each group.

**Table 2:** Changes of lipid and glucose markers after the load test

	Before	After			
		0h	2h	4h	6h
*Triglyceride (mg/dL)*					
Non-diabetic group (*n* = 32)	122.7 ± 40.5	125.7 ± 43.4	163.2 ± 65.4	232.2 ± 71.8^†^	215.7 ± 64.6^†^
Prediabetic group (*n* = 47)	141.2 ± 74.7	146.9 ± 78.5	198.5 ± 84.5	287.6 ± 110.9^‡^	291.2 ± 135.6^‡^
Diabetic group (*n* = 33)	140.3 ± 76.8	139.2 ± 82.7	224.3 ± 85.2^¶^	355.9 ± 114.7^¶^	364.8 ± 127.3^¶^
*LDL cholesterol (mg/dL)*					
Non-diabetic group (*n* = 32)	95.8 ± 27.9	97.4 ± 26.7	91.0 ± 26.8	89.2 ± 24.4	91.0 ± 26.2
Prediabetic group (*n* = 47)	100.5 ± 30.4	101.4 ± 32.6	95.3 ± 29.3	93.8 ± 29.3	94.8 ± 27.6
Diabetic group (*n* = 33)	100.5 ± 20.1	101.5 ± 20.1	94.5 ± 18.4	91.9 ± 18.1	93.9 ± 18.8
*HDL cholesterol (mg/dL)*					
Non-diabetic group (*n* = 32)	46.5 ± 11.5	48.0 ± 12.3	44.6 ± 11.7	43.5 ± 10.5	44.5 ± 11.1
Prediabetic group (*n* = 47)	51.9 ± 12.7	53.1 ± 11.6	50.1 ± 11.3	48.7 ± 10.4	49.8 ± 11.8
Diabetic group (*n* = 33)	55.6 ± 16.8	56.4 ± 17.6	52.4 ± 16.5	51.3 ± 16.3	52.0 ± 17.0
*RLP cholesterol (mg/dL)*					
Non-diabetic group (*n* = 32)	4.3 ± 1.3	4.4 ± 1.4	5.7 ± 2.2^‡^	8.2 ± 2.4^‡^	7.6 ± 2.2^†^
Prediabetic group (*n* = 47)	4.9 ± 2.5	5.1 ± 2.7	7.0 ± 2.9^†^	10.1 ± 3.9^§^	10.3 ± 4.7^§^
Diabetic group (*n* = 33)	4.9 ± 2.6	4.9 ± 2.8	7.9 ± 2.9^¶^	14.6 ± 4.0^¶^	16.9 ± 4.4^¶^
*MDA LDL (U/L)*					
Non-diabetic group (*n* = 32)	98.7 ± 36.0	105.7 ± 38.0	93.2 ± 28.0	89.0 ± 27.4	90.3 ± 33.6
Prediabetic group (*n* = 47)	107.3 ± 28.6	112.5 ± 31.4	100.1 ± 30.0	101.9 ± 32.0	104.2 ± 32.3
Diabetic group (*n* = 33)	114.3 ± 32.6	117.4 ± 33.3	104.0 ± 26.8	103.3 ± 28.8	109.1 ± 28.9
*Apolipoprotein A-I (mg/dL)*					
Non-diabetic group (*n* = 32)	121.7 ± 19.0	125.5 ± 20.6	118.2 ± 19.1	118.3 ± 15.1	119.5 ± 17.7
Prediabetic group (*n* = 47)	137.5 ± 26.6	139.5 ± 27.5	132.2 ± 24.0	132.0 ± 22.2	132.2 ± 24.7
Diabetic group (*n* = 33)	139.7 ± 27.8	141.9 ± 33.2	135.4 ± 32.0	135.3 ± 31.3	136.3 ± 33.1
*Apolipoprotein B (mg/dL)*					
Non-diabetic group (*n* = 32)	82.5 ± 18.8	84.2 ± 18.4	79.2 ± 18.0	80.2 ± 17.2	80.3 ± 18.2
Prediabetic group (*n* = 47)	85.0 ± 22.4	86.7 ± 22.9	82.0 ± 20.9	82.4 ± 22.2	82.3 ± 21.1
Diabetic group (*n* = 33)	86.9 ± 15.7	87.4 ± 15.5	83.5 ± 14.1	84.0 ± 14.3	84.8 ± 14.1
*Glucose (mg/dL)*					
Non-diabetic group (*n* = 32)	90.5 ± 14.3	113.3 ± 21.1^¶^	101.1 ± 28.7	95.5 ± 11.5	94.0 ± 5.7
Prediabetic group (*n* = 47)	103.8 ± 14.6	121.9 ± 22.4^¶^	123.8 ± 31.9^¶^	97.1 ± 15.7	97.7 ± 7.6^†^
Diabetic group (*n* = 33)	138.7 ± 9.6	158.4 ± 36.2	182.0 ± 35.7^†^	129.0 ± 26.1	117.5 ± 19.8
*Insulin (μIU/mL)*					
Non-diabetic group (*n* = 32)	6.1 ± 3.1	21.8 ± 6.1^§^	23.7 ± 7.1^§^	14.0 ± 7.8^†^	6.4 ± 4.5
Prediabetic group (*n* = 47)	6.4 ± 3.8	20.6 ± 12.3^¶^	28.1 ± 7.6^¶^	13.3 ± 6.9^¶^	7.6 ± 4.3
Diabetic group (*n* = 33)	8.4 ± 3.7	24.7 ± 8.5^¶^	33.8 ± 7.9^¶^	20.8 ± 8.8^¶^	10.5 ± 6.9

Values are shown as mean ± SD

*LDL* low-density lipoprotein, *HDL* high-density lipoprotein, *RLP* remnant lipoprotein, *MDA* malondialdehyde-modified

^†^ *P* <0.05, ^‡^ *P* <0.01, ^§^ *P* <0.005, ^¶^ *P* < 0.001 comparing with the value before the load test in the same group

**Fig. 1:**
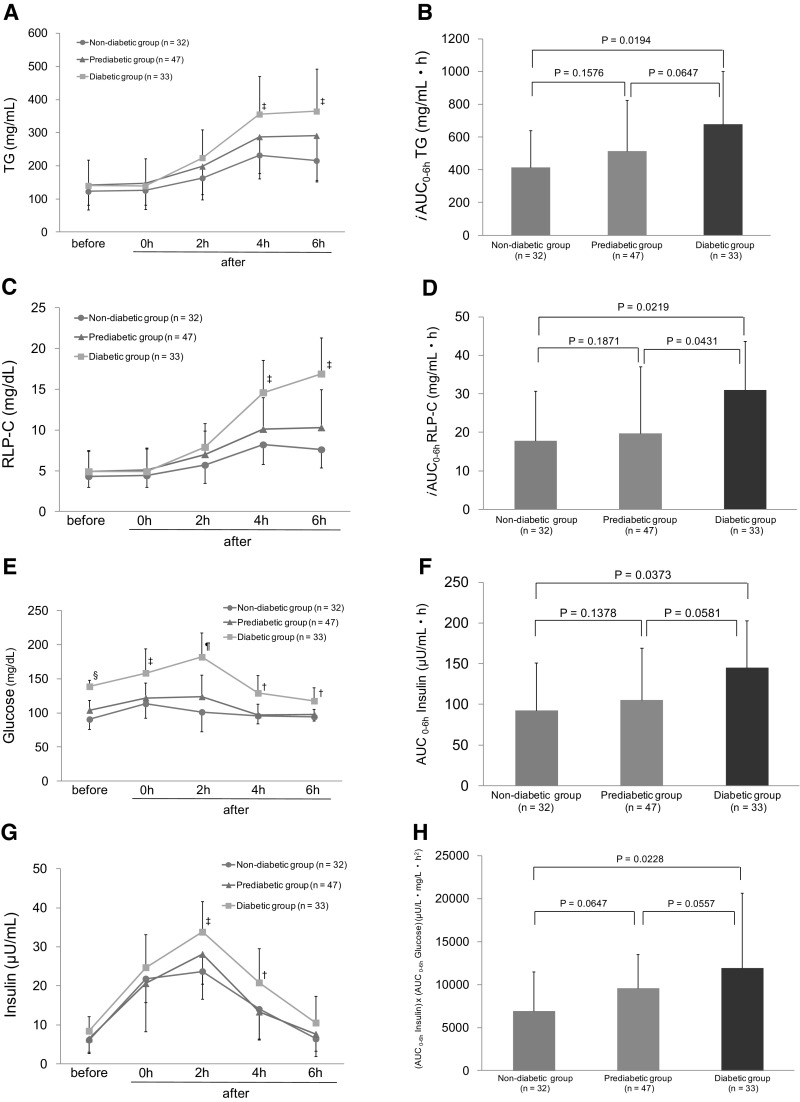
Postprandial changes in serum TG (**a**), RLP-C(C), glucose (**e**), and insulin (**g**) levels after the fat loading test; comparison of *i*AUCs for postprandial serum TG (**b**), RLP-C(D); comparison of AUCs for plasma insulin (**f**) levels and insulin resistance index (**h**) during the test. Data are expressed as mean ± SD. ^†^ *P*  < 0.05, ^‡^ *P*  < 0.01, ^§^ *P* < 0.005, ^¶^ *P* < 0.001 comparing with the value at the same time points in non-diabetic CAD group. *TG* triglycerides, *RLP-C* remnant-like particle cholesterol, *AUC* area under the curve, *iAUC* incremental area under the curve. Insulin resistance index is one of the insulin resistance markers as calculated by (AUCs of insulin) × (AUCs of glucose)

Figure 1a shows the changes in serum TG levels after the fat loading test in three groups. The values of serum TG in diabetic group at the 4th and 6th hour were significantly higher than those in non-diabetic group (4th hour: 355.9 ± 114.7 vs. 232.2 ± 71.8 mg/dL, *P* = 0.0083; 6th hour: 364.8 ± 127.3 vs. 215.7 ± 64.6 mg/dL, *P* = 0.0061). There were no significant differences in serum TG values at the 4th and 6th hour between diabetic and prediabetic CAD group (4th hour: 355.9 ± 114.7 vs. 287.6 ± 110.9 mg/dL, *P* = 0.0847; 6th hour: 364.8 ± 127.3 vs. 291.2 ± 135.6 mg/dL, *P* = 0.1864). There were also no significant differences in serum TG values at the 4th and 6th hour between prediabetic and non-diabetic CAD group (4th hour: 287.6 ± 110.9 vs. 232.2 ± 71.8 mg/dL, *P* = 0.1184; 6th hour: 291.2 ± 135.6 vs. 215.7 ± 64.6 mg/dL, *P* = 0.0749).

The *i*AUCs of serum TG levels in diabetic CAD group were significantly greater than those in non-diabetic CAD group (*P* = 0.0194). There were no significant differences in *i*AUCs of serum TG levels between diabetic and prediabetic CAD group (*P* = 0.0647), or prediabetic and non-diabetic CAD group (*P* = 0.1576) (Fig. 1b).

As shown in Fig. 1c, the changes in serum RLP-C levels after the fat loading test for three groups were similar to those in serum TG levels. The values of serum RLP-C levels in diabetic CAD group at the 4th and 6th hour were significantly higher than those in non-diabetic CAD group (4th hour: 14.6 ± 4.0 vs. 8.2 ± 2.4 mg/dL, *P* = 0.0064; 6th hour: 16.9 ± 4.4 vs. 7.6 ± 2.2 mg/dL, *P* = 0.0057). There were no significant differences in serum RLP-C levels at the 4th and 6th hour between prediabetic and diabetic or non-diabetic CAD group. The *i*AUCs of serum RLP-C levels in diabetic CAD group were significantly greater than those in prediabetic and non-diabetic CAD groups (*P* = 0.0431, 0.0219, respectively). There were no significant differences in *i*AUCs of serum RLP-C levels between prediabetic and non-diabetic CAD group (*P* = 0.1871) (Fig. 1d).

As shown in Fig. 1e, g, the serum glucose and insulin levels in each group increased postprandially and reached peak levels at the 2nd hour, and then returned to baseline levels by the 4th or 6th hour after the test. Compared with non-diabetic CAD group, diabetic CAD group showed that the plasma insulin levels at the 2nd hour after the test were significantly higher (*P* = 0.0067) (Fig. 1g). The AUCs of plasma insulin levels as the insulin resistance marker were greater in diabetic CAD group than in non-diabetic CAD group (145.3 ± 57.9 vs. 92.6 ± 58.7, *P* = 0.0373) (Fig. 1f). The insulin resistance index: (AUCs of insulin) × (AUCs of glucose) was also greater in diabetic CAD group than non-diabetic CAD group (11,943 ± 8730 vs 6938 ± 4587, *P* = 0.0228) (Fig. 1h).

### Relationship between lipid markers and insulin resistance markers

The data on insulin resistance markers in each group are summarized in Table 3. There were significant differences in insulin resistance markers except fasting plasma insulin levels among three groups, and diabetic CAD group had higher levels of insulin resistance than non-diabetic CAD group (HOMA-IR, 2.1 ± 1.3 vs. 1.2 ± 0.9, *P* = 0.0075; insulin levels at the 2nd hour, 33.8 ± 8.5 vs. 23.7 ± 7.1, *P* = 0.0183).

**Table 3:** Insulin resistance markers

	Non-diabetic CAD group (*n* = 32)	Prediabetic CAD group (*n* = 47)	Diabetic CAD group (*n* = 33)
HOMA-IR	1.2 ± 0.9	1.7 ± 1.0	2.1 ± 1.3^‡^
Fasting insulin (µIU/mL)	6.1 ± 3.1	6.4 ± 3.8	8.4 ± 3.7
2 h insulin (µIU/mL)	23.7 ± 7.1	28.1 ± 7.6	33.8 ± 7.9^‡^
AUC_0–6h_ insulin (µIU/mL h)	92.6 ± 58.7	105.1 ± 64.3	145.3 ± 57.9^†^
AUC insulin × AUC glucose (µIU/L mg/L h^2^)	6938 ± 4587	9573 ± 3942	11943 ± 8730^†^

Values are shown as mean ± SD

*HOMA-IR* homeostasis model assessment of insulin resistance, *AUC* area under the curve

^†^ *P* < 0.05, ^‡^ *P* < 0.01 comparing with the value in non-diabetic group

**Fig. 2:**
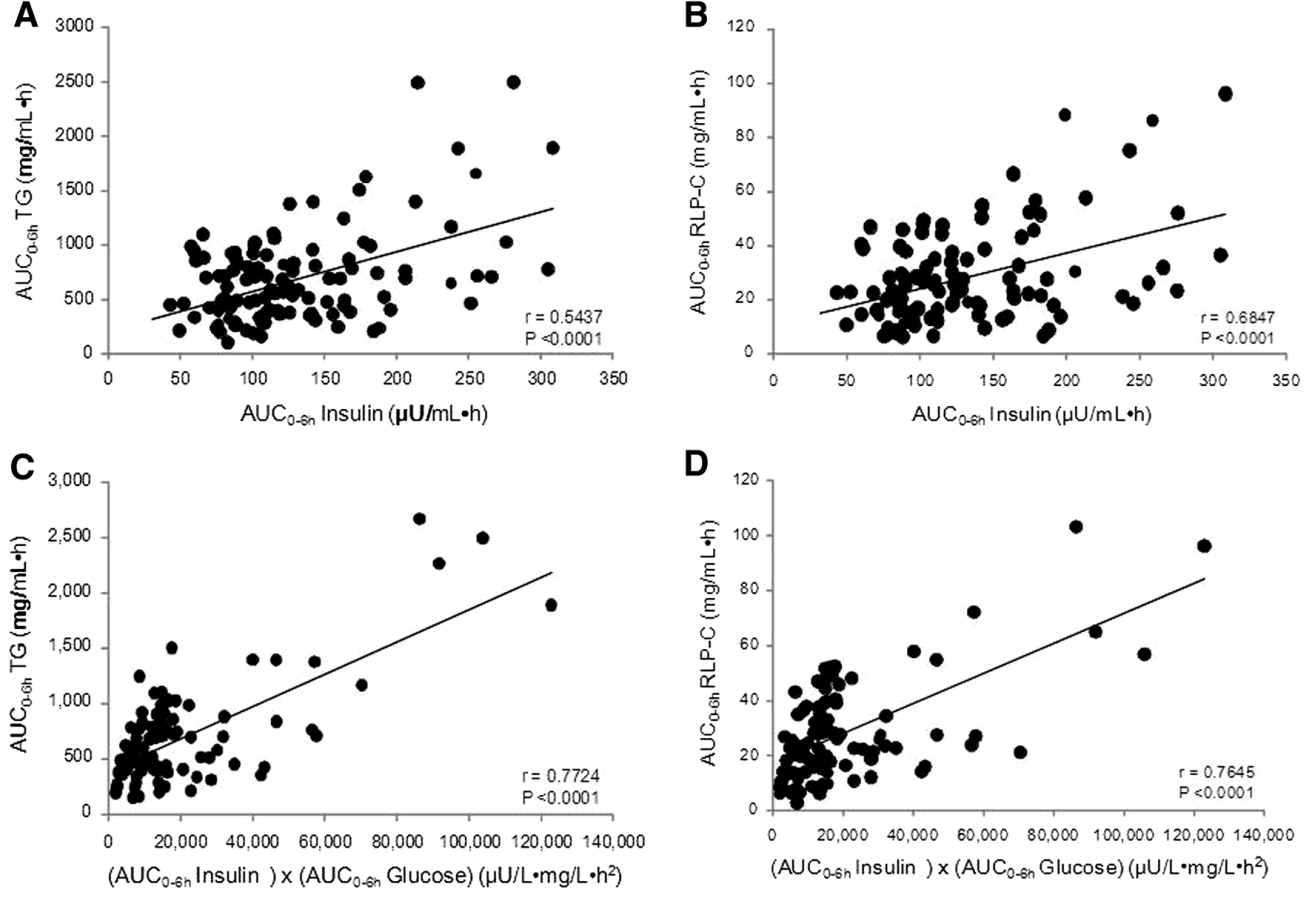
Correlations between the value of AUCs for postprandial serum TG or RLP-C levels and those for postprandial plasma insulin or insulin resistance index (IRI) in CAD groups. **a** TG vs insulin, **b** RLP-C vs insulin, **c** TG vs IRI, **d** RLP-C vs IRI. *TG* triglycerides, *RLP-C* remnant-like particle cholesterol, *AUC* area under the curve. Insulin resistance index is one of the insulin resistance markers as calculated by (AUCs of insulin) × (AUCs of glucose)

Figure 2 shows the relationship between lipid markers and insulin resistance markers in the cake sále test. The AUCs of serum TG or RLP-C levels showed a correlation with the AUCs of plasma insulin (AUC-TG, *r* = 0.5437, *P* < 0.0001; AUC-RLC-P, *r* = 0.6847, *P* < 0.0001) (Fig. 2a, b), and furthermore, they correlated well with the insulin resistance index: (AUCs of insulin) × (AUCs of glucose) (AUC-TG, *r* = 0.7724, *P* < 0.0001; AUC-RLC-P, *r* = 0.7645, *P* < 0.0001) (Fig. 2c, d). There did not exist a definite correlation between insulin resistance markers and such lipid markers as HOMA-IR, fasting insulin, insulin levels at the 2nd hour (data not shown).

## Discussion

The main findings of the present study were that (1) the magnitude of postprandial serum TG or RLP-C accumulation, which was shown as the area under the TG or RLP-C curve over 6 h after the fat loading test, was greater in diabetic CAD group than in non-diabetic or prediabetic CAD group, and (2) it correlated well with insulin resistance markers: the AUCs of insulin and the insulin resistance index described as [(AUCs of insulin) × (AUCs of glucose)].

Hyperlipidemia has been established as a major independent risk factor for CAD and therapies aimed at reducing serum LDL-C level are considered to be an essential element of any attempt to prevent CAD; however, the role of hypertriglyceridemia is not fully understood. Not only fasting but also non-fasting hypertriglyceridemia have shown as an important factor in the development of atherosclerosis and closely related to the occurrence of cardiovascular events [3]. Non-fasting, postprandial hypertriglyceridemia is characterized by accumulation of excess TG-rich lipoproteins and their partially hydrolyzed products such as chylomicron (CM) remnant (CM-R) and very low-density lipoprotein (VLDL) remnant (VLDL-R) during the postprandial period. For estimation of the risk for CAD, the measurement of lipid parameters such as serum TG and RLP-C may be more important in non-fasting state such as postprandial condition than in fasting state. Although postprandial hyperlipidemia has been reported to be a predisposing factor in coronary events [3, 4], little information has been available regarding the observation of postprandial kinetic responses to a fat load in CAD patients confirmed by angiography. In the present study, the levels of serum TG and RLP-C kept rising during 6 h after the load and the kinetics of these serum levels were similar among three CAD groups. These results were compatible with the previous reports which were obtained from the relatively small number of CAD patients [13, 14].

Dyslipidemia characterized by high levels of serum fasting TG and low levels of serum HDL-C is common in patients with T2DM [15]. Moreover, it has been known that these patients showed high and prolonged postprandial lipidemia after meals [16]. In the present study, CAD patients were divided into three groups (non-diabetic, prediabetic, diabetic groups), depending on the degree of diabetic condition and then the comparisons were made of postprandial lipid and glucose metabolism. Although the different responses of serum TG or RLP-C levels after the load were shown between patients with T2DM and normal control subjects [15], there is limited information on postprandial lipid metabolism in different diabetic condition. To our knowledge, this is the first report as to the postprandial lipid responses in CAD patients, depending on different diabetic conditions. It was found that the magnitude of postprandial serum TG or RLP-C accumulation was significantly greater in diabetic CAD group when compared with prediabetic or non-diabetic CAD group, whereas it was no different between prediabetic and non-diabetic CAD group. Although we analyzed serum RLP-C levels after the load, the measurement of remnant lipoprotein cholesterol (RemL-C) which is more direct marker for CM-R and VLDL-R might provide valuable additional information in the present study.

It has been hypothesized that two pathways (endogenous and exogenous pathways) could induce the postprandial lipid accumulations in patients with T2DM. Endogenous pathway is characterized by an increased hepatic VLDL production, as a result from increased free-fatty acid (FFA) release from adipose tissue and inefficient suppression of hepatic VLDL release by insulin. Patients with T2DM has been reported to show the diminution of antilipolytic effect by insulin, leading to a higher FFA flux from adipose tissue [17]. Exogenous pathway is closely related with an increase of lipid absorption from the intestine in patients with T2DM. Animal models using streptozotocin-induced diabetic rats showed an increased intestinal TG production and an increased intestinal absorption of cholesterol [18]. In patients with T2DM, the absorption of cholesterol was shown to be higher in CAD patients than non-CAD patients [19]. Epidemiological study also demonstrated that impaired cholesterol homeostasis, reflected by lower synthesis markers such as lathosterol and higher absorption markers such as campesterol, was a highly significant independent predictor of CAD in the Framingham Offspring Study Cycle-6 participants including approximately 30 % of patients with DM [20]. Which pathway could contribute significantly to the elevated lipid levels after the meal in CAD patients with T2DM? Recently, Masuda et al. [21] measured Apo B-48 and Apo B-100 levels separately in fasting and postprandial state to assess the endogenous and exogenous pathway independently, and they reported that postprandial increase in serum TG and RLP-C levels was mainly due to increase of CM and CM-R, but not VLDL and VLDL-R. Unfortunately, we could not answer the above question in the present study because we could not distinguish between the changes of endogenous pathway and those of exogenous pathway. Measurement of not only serum TG or RLP-C levels but also lipid markers related with exogenous pathway such as Apo B-48, CM, or CM-R would contribute to better understanding of the pathophysiological differences in the exogenous versus endogenous pathway.

Our results showed that the postprandial responses of lipid markers were similar between pre- and non-diabetic CAD group, and that the magnitude of postprandial serum TG or RLP-C accumulation was no significantly different between these two groups. In the present study, prediabetic state was defined as impaired fasting glucose (IFG) by FPG levels (100–125 mg/dL) and HbA1c levels (5.7–6.4 %) according to 2010 ADA Guidelines [8], not as impaired glucose tolerance (IGT) by 2-h 75 g oral glucose tolerance test (OGTT) values which could affect glycemic state or insulin resistance as accurately as possible. Addition of the estimation by OGTT would provide more useful information about the difference of postprandial lipid metabolism between prediabetic patients with and without IGT in the present study; however, there was a need to simplify screening test for glycemic state so relatively large number of patients could be identified earlier and more efficiently.

Not only impaired postprandial lipid metabolism but also hyperinsulinemia has been thought to play an important role in the development of atherosclerosis [22, 23]. The postprandial increase of plasma insulin levels after the load was shown to be greater in diabetic CAD group than non-diabetic or prediabetic CAD group. And the sum of the amount of increase of plasma insulin values during the test was significantly higher in diabetic CAD group as compared with non-diabetic or prediabetic CAD group. These results suggest that diabetic CAD patients show higher insulin resistant compared with non- or prediabetic CAD patients. There is clinical evidence to indicate that metformin which is an oral antidiabetic drug in the biguanide class [24], pioglitazone which is an agonist of peroxisome proliferator-activated receptor [25], or *α*-glucosidase inhibitor which inhibits the absorption of carbohydrates in the gastrointestinal tract [26], reduce the risk of cardiovascular events in T2DM patients. The anti-atherosclerotic effect of these drugs which reduce insulin resistance might be produced partially via improvement of postprandial hyperinsulinemia and/or hyperlipidemia.

### Study limitations

There are several limitations to the present study. First, all subjects of the present study were male patients to exclude the hormonal effect by estrogen on postprandial lipid metabolism. Second, the present study did not include the patients with insulin therapy for excluding the effects by exogenous insulin. The postprandial lipid metabolism remains unknown in these patients. Third, all patients in CAD group were treated with statins. Because the possibility exists that the effective inhibition of hepatic cholesterol synthesis by statins leads to the increase of intestinal absorption of cholesterol [27], more investigation for cholesterol absorption/synthesis markers such as cholesterol or lathosterol [28] would provide valuable additional information in the present study.

## Conclusions

We examined the relationship between the severity of glycemic state and the degree of postprandial lipidemic responses in CAD patients. Our results suggest that insulin resistance shows a close relationship with postprandial hyperlipidemia, and that diabetic state, but not prediabetic state, may be a risk for postprandial impaired lipid metabolism.
